# Association between cathepsins and benign prostate diseases: a bidirectional two-sample Mendelian randomization study

**DOI:** 10.3389/fendo.2024.1348310

**Published:** 2024-06-05

**Authors:** Hongliang Cao, Bin Liu, Kejian Gong, Hao Wu, Yishu Wang, Haiyang Zhang, Chengdong Shi, Pengyu Wang, Hao Du, Honglan Zhou, Song Wang

**Affiliations:** ^1^ Department of Urology II, The First Hospital of Jilin University, Changchun, China; ^2^ Department of Thoracic Surgery, The First Hospital of Jilin University, Changchun, China; ^3^ Key Laboratory of Pathobiology, Ministry of Education, Jilin University, Changchun, China; ^4^ Department of Prosthodontics, Hospital of Stomatology, Jilin University, Changchun, China

**Keywords:** cathepsins, benign prostate diseases, Mendelian randomization, bi-directional causal effects, benign prostatic hyperplasia (BPH)

## Abstract

**Objectives:**

The relationship between cathepsins and prostate cancer (PCa) has been reported. However, there is a lack of research on cathepsins and benign prostate diseases (BPDs). This study investigated the potential genetic link between cathepsins and BPDs through the utilization of Mendelian randomization (MR) analysis to determine if a causal relationship exists.

**Methods:**

Publicly accessible summary statistics on BPDs were obtained from FinnGen Biobank. The data comprised 149,363 individuals, with 30,066 cases and 119,297 controls for BPH, and 123,057 individuals, with 3,760 cases and 119,297 controls for prostatitis. The IEU OpenGWAS provided the Genome-wide association data on ten cathepsins. To evaluate the causal relationship between BPDs and cathepsins, five distinct MR analyses were employed, with the primary method being the inverse variance weighted (IVW) approach. Additionally, sensitivity analyses were conducted to examine the horizontal pleiotropy and heterogeneity of the findings.

**Results:**

The examination of IVW MR findings showed that cathepsin O had a beneficial effect on BPH (IVW OR=0.94, 95% CI 0.89–0.98, P=0.0055), while cathepsin X posed a threat to prostatitis (IVW OR=1.08, 95% CI 1.00–1.16, P=0.047). Through reverse MR analysis, it was revealed that prostatitis had an adverse impact on cathepsin V (IVW OR=0.89, 95% CI 0.80–0.99, P=0.035), while no favorable association was observed between BPH and cathepsins. The results obtained from MR-Egger, weighted median, simple mode, and weighted mode methods were consistent with the findings of the IVW approach. Based on sensitivity analyses, heterogeneity, and horizontal pleiotropy are unlikely to distort the results.

**Conclusion:**

This study offers the initial evidence of a genetic causal link between cathepsins and BPDs. Our findings revealed that cathepsin O was beneficial in preventing BPH, whereas cathepsin X posed a potential threat to prostatitis. Additionally, prostatitis negatively affected cathepsin V level. These three cathepsins could be targets of diagnosis and treatment for BPDs, which need further research.

## Introduction

Benign prostatic diseases (BPDs), mainly including benign prostatic hyperplasia (BPH) and prostatitis, are prevalent urological ailments affecting males globally (1, 2). Compression of the urethra caused by BPH results in clinical symptoms that impact the quality of life for individuals with BPH, including lower urinary tract symptoms (LUTS), episodes of acute urinary retention, and recurring urinary infections (3). Numerous investigations have been carried out regarding the development of BPH; nevertheless, the factors responsible for its occurrence and advancement are still poorly understood (4). Prostatitis is classified into types I–IV by the National Institutes of Health (NIH) in the United States. Chronic prostatitis/chronic pelvic pain syndrome (CP/CPPS) of Type III is categorized into inflammatory CPPS (IIIa) and noninflammatory CPPS (IIIb), making up approximately 90%–95% of cases related to prostatitis (5). Individuals diagnosed with CP/CPPS typically encounter discomfort in the pelvic region, such as pain in the pubic region or perineum, discomfort during sexual activity or ejaculation, painful urination, frequent urination at night and/or a sense of urgency, sexual difficulties, impotence, mental health disorders, and reduced sperm quality (6, 7). Exploring the etiology, diagnosis, and treatment of BPDs in public health has significant practical implications by identifying the causal association of risk factors that can be modified.

The group of lysosomal proteolytic enzymes known as cathepsins plays a crucial role in preserving cellular homeostasis, which are classified into multiple families, including serine proteases (cathepsins A and G), cysteine proteases (cathepsins B, C, F, H, K, L, O, S, V, W, and X), and aspartyl proteases (cathepsins D and E) (8, 9). They are associated with many cellular activities including protein and lipid metabolism, autophagy, antigen presentation, growth factor receptor recycling, cellular stress signaling, extracellular matrix degradation, and lysosome-mediated cell death (10). Various cathepsins have significant roles in various diseases due to their participation in these essential processes (11). The association between cathepsins and prostate cancer (PCa) has been reported, such as cathepsin B. Nalle et al. found that the inhibition of invasion and migration and the activation of apoptosis in PC3 and DU145 prostate cancer cell lines can be achieved by targeting cathepsin B (12). Moreover, the rise in serum levels of cathepsin B and the density of cathepsin B may serve as innovative indicators for the progression of the disease. However, they do not impact the survival of individuals diagnosed with PCa (13). Furthermore, the generation of sphingosine 1-phosphate through acid ceramidase facilitates the invasion of PCa by increasing the expression of cathepsin B (14). Despite several studies suggesting potential applications of cathepsins in PCa disease, there is a lack of studies exploring cathepsins in relation to BPDs. Based on this, we will use Mendelian randomization (MR) to do such a thing.

MR designs utilize genetic diversity as instrumental variables (IVs) to explore the causal association between potential factors and the specific disease, offering several advantages absent in conventional epidemiology (15). In addition, using MR designs can decrease the likelihood of reverse causality and minimize the impact of confounding variables (16). The objective of this study was to examine the causal connection between cathepsins and BPDs, potentially identifying novel avenues for diagnosing and treating BPDs.

## Materials and methods

### Study design

**Figure 1:**
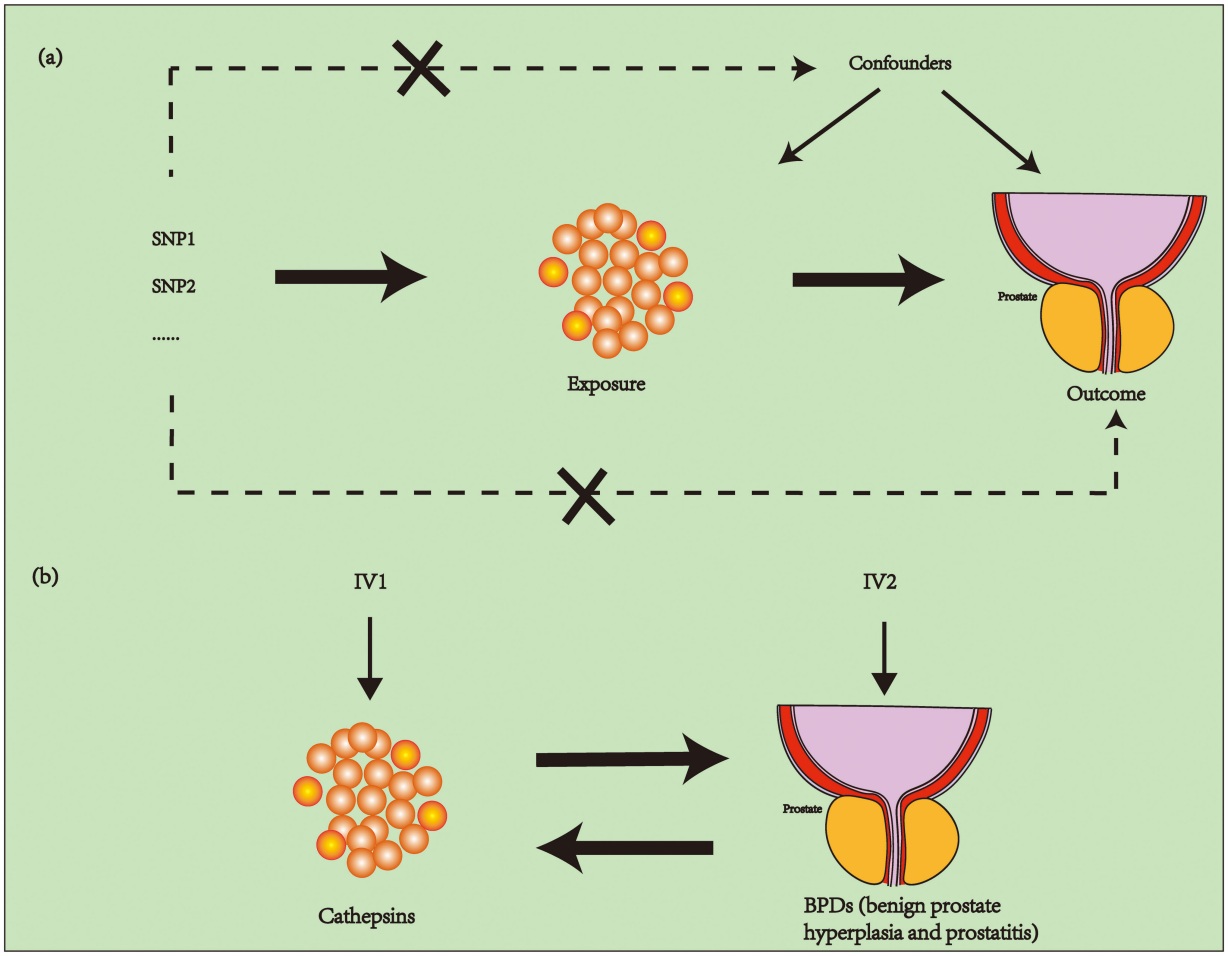
The schematic of an MR study involving cathepsins and BPDs (BPH and prostatitis) using two samples. **(A)** In order for the MR study to be valid, three essential assumptions must be met: i) relevance assumption: the IVs must be linked to the exposure; ii) independence assumption: the IVs should not be correlated with any confounding factors; and iii) exclusion assumption: the IVs must solely impact the outcome through the exposure. **(B)** We explore the bidirectional two-sample MR between cathepsins and BPDs. MR, Mendelian randomization; BPDs, benign prostatic diseases; BPH, benign prostatic hyperplasia; IVs, instrumental variables.

To establish the bidirectional causal connection between BPDs and cathepsins, a Mendelian randomization (MR) study using two samples was conducted. To investigate the causal relationship between exposure and outcome, the research on MR utilizes instrumental variables (IVs) and examines Single Nucleotide Polymorphisms (SNPs). To acquire valid IVs, three essential assumptions must be met: i) relevance assumption: the IVs must be strongly linked to the exposure (cathepsins); ii) independence assumption: the IVs should not be correlated with any confounding factors; and iii) exclusion assumption: the IVs must solely impact the outcome (BPDs) through the exposure (cathepsins) (17, 18). **Figure 1** shows the planned layout of bidirectional two-sample MR between cathepsins and BPDs.

### GWAS statistics source

Summary statistics on BPDs obtained from FinnGen Biobank (website: https://www.finngen.fi/en) were utilized. The dataset included 149,363 (30,066 cases and 119,297 controls) and 123,057 (3,760 cases and 119,297 controls) separately for BPH and prostatitis. BPH cases are defined in the database based on the N40 criteria in the International Classification of Diseases 10 (ICD-10) Revision codes, as well as the codes 600 in ICD-8 and ICD-9. Prostatitis is a condition that causes inflammation in the prostate gland, which can be caused by infection or non-infectious factors. The objective of FinnGen Biobank, a collaboration between the public and private sectors, is to gather and examine genetic and medical information from 500,000 individuals involved in the Finnish biobank (19). The IEU OpenGWAS (https://gwas.mrcieu.ac.uk) provided the ten Genome-wide association studies (GWAS) of cathepsins (20, 21). The details of GWAS included in MR analyses are described in **Table 1**.

**Table 1:** Details of GWAS included in MR analyses.

Trait	IEU GWAS ID	Consortium	Ethnicity	Sample size
BPH	–	FinnGen Biobank	European	149363
Prostatitis	–	FinnGen Biobank	European	123057
Cathepsin S	prot-a-727	NA	European	3301
Cathepsin F	prot-a-722	NA	European	3301
Cathepsin G	prot-a-723	NA	European	3301
Cathepsin H	prot-a-725	NA	European	3301
Cathepsin B	prot-a-718	NA	European	3301
Cathepsin O	prot-a-726	NA	European	3301
Cathepsin E	prot-a-720	NA	European	3301
Cathepsin X	prot-a-729	NA	European	3301
Cathepsin V	prot-a-728	NA	European	3301
Cathepsin D	prot-b-51	NA	European	3394

BPH, benign prostate hyperplasia. NA, Not acquired. “-“ indicates not applicable.

### Selection of genetic instrumental variables

In order to obtain effective IVs, we selected SNPs that were highly correlated with exposure. A conventional threshold was considered, with significant genome-wide importance (P<5× 10^-8^), linkage disequilibrium (LD), and an r^2^ less than 0.001 within a 10,000 kb range. Consequently, the number of SNPs is insufficient to perform MR, thus the following criteria were adopted. We identified SNPs that showed genome-wide significance (P<5×10^-6^, and P<5×10^-8^ for BPH in reverse MR), LD, and an r^2^<0.001 threshold within a 10,000 kb range. We removed palindromic variants for incompatible alleles. The F-statistics were calculated utilizing the following equation:


$$F=\frac{R^{2}\left(N-1-K\right)}{\left(1-R^{2}\right)K}$$

R^2^ represents the proportion of variance in exposure that can be accounted for by the IVs, N is the sample size, and K is the number of IVs. SNPs were considered for inclusion if the F-statistic was more significant than or equal to 10, indicating a strong potential to predict exposure (22). The Steiger test posits that the correlation between SNPS and exposure should be greater than the outcome, otherwise reverse causality will occur, and SNPS that fail the test may be unrelated to exposure and excluded (23). MR-PRESSO is capable of detecting horizontal pleiotropy and correcting it by removing outliers. Furthermore, it is able to test the significance of causal inference both before and after the aforementioned correction (24). The chosen SNPs were the final instrumental variables (IVs) for the following MR study.

### MR analyses and sensitivity analyses

**Figure 2:**
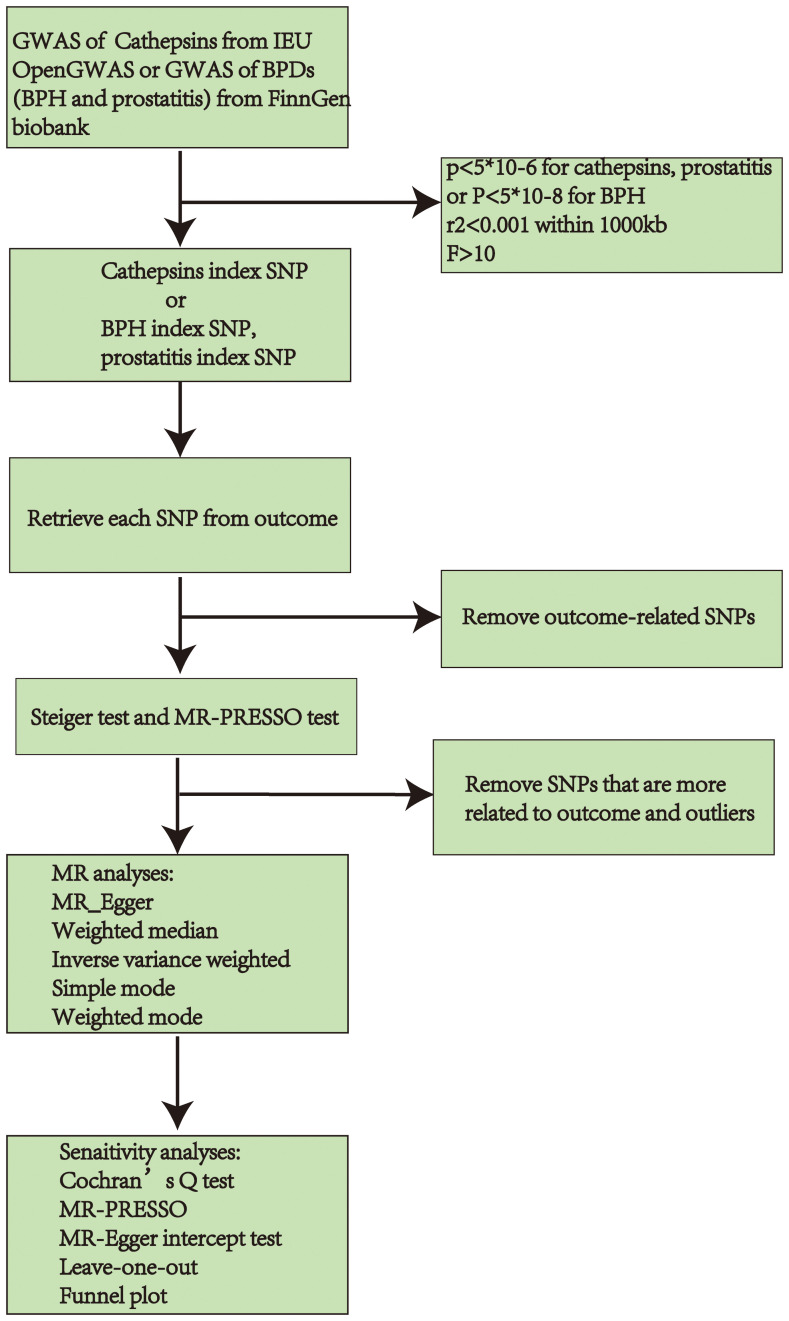
Workflow chart of MR discovering causality between cathepsins and BPDs (BPH and prostatitis). GWAS, Genome-Wide Association Study; BPDs, benign prostate diseases; BPH, benign prostate hyperplasia; SNP, Single Nucleotide Polymorphisms; MR, Mendelian randomization.

For MR analyses, five MR methods were employed, namely MR Egger, weighted median, inverse-variance weighted (IVW), simple mode, and weighted mode. The IVW estimates were selected as the primary method, and four additional methods were used to improve their robustness in a broader range of scenarios (25). Cochran’s Q test of IVW and MR Egger (26) also identified heterogeneity. Additionally, the Pleiotropy Residual Sum and Outlier methods (MR-PRESSO) were employed to evaluate and rectify horizontal pleiotropy (24). Moreover, a Leave-one-out analysis was conducted to assess if a solitary SNP influenced or skewed the MR estimate. A funnel plot was employed to assess the likely directional pleiotropy. A statistical significance was determined when the significance level was below 0.05. **Figure 2** displays the described workflow of MR and sensitivity analyses. The statistical analyses were conducted in R software 4.3.1 using the TwoSampleMR R package (version 0.5.7) and MR-PRESSO (version 1.0).

## Results

**Table 2** displays the results of bidirectional two-sample MR between cathepsins and BPDs, and we found three statistically significant positive results. By utilizing the 12 SNPs associated with cathepsin O, we discovered that elevated levels of cathepsin O can decrease the risk of BPH (OR=0.94, 95%CI=0.89–0.98, p-value=0.00553) through the implementation of IVW techniques (**Table 2**; **Supplementary Table 1**; **Supplementary Figure 1**). Moreover, the absence of significant heterogeneity (P-value > 0.05) was confirmed by the MR-Egger and IVW heterogeneity tests. The absence of any impact was shown when using P-values obtained from MR-Pleiotropy Residual Sum and Outlier methods (MR-PRESSO) to evaluate horizontal pleiotropy (**Table 3**; **Supplementary Table 2**). In the Leave-one-out sensitivity analysis (**Supplementary Figure 2A**), there was no individual SNP that significantly undermined the overall impact of cathepsin O on BPH. The symmetrical funnel plot also suggests the absence of pleiotropy (**Supplementary Figure 2B**). Furthermore, by employing 12SNPs, we discovered a correlation between elevated cathepsin X levels and heightened susceptibility to prostatitis (OR=1.08, 95% CI=1.00–1.16, p-value=0.0477) through the implementation of IVW techniques (**Table 2**; **Supplementary Table 3**; **Supplementary Figure 3**). Comparable to the findings mentioned above, there was an absence of heterogeneity or horizontal pleiotropy in the outcomes (P-value > 0.05) (**Table 3**; **Supplementary Table 4**; **Supplementary Figure 4**).

**Table 2:** Results of bidirectional two-sample MR between cathepsins and BPDs.

	MR			Reverse MR		
Cathepsin	SNPs	Inverse variance weighted		SNPs	Inverse variance weighted	
		OR (95%CI)	p_value		OR (95%CI)	p_value
Cathepsin S						
BPH	20	1.00 (0.97–1.03)	0.983	35	0.92 (0.82–1.04)	0.174
Prostatitis	20	0.99 (0.92–1.06)	0.773	15	0.94 (0.84–1.05)	0.281
Cathepsin F						
BPH	11	1.00 (0.97–1.04)	0.829	35	0.96 (0.86–1.09)	0.539
Prostatitis	11	0.99 (0.91–1.08)	0.815	15	1.00 (0.89–1.12)	0.937
Cathepsin G						
BPH	9	1.01 (0.94–1.08)	0.881	35	1.02 (0.91–1.15)	0.719
Prostatitis	9	1.09 (0.93–1.28)	0.295	15	1.01 (0.91–1.12)	0.879
Cathepsin H						
BPH	7	1.00 (0.97–1.02)	0.826	35	1.02 (0.91–1.15)	0.725
Prostatitis	7	1.03 (0.97–1.09)	0.315	15	0.94 (0.85–1.04)	0.217
Cathepsin B						
BPH	17	1.02 (0.99–1.06)	0.238	35	0.98 (0.86–1.11)	0.713
Prostatitis	17	1.03 (0.95–1.12)	0.433	15	0.98 (0.88–1.08)	0.661
Cathepsin O						
BPH	12	0.94 (0.89–0.98)	0.00553	35	1.01 (0.90–1.14)	0.840
Prostatitis	12	1.05 (0.94–1.17)	0.405	15	0.93 (0.82–1.06)	0.278
Cathepsin E						
BPH	8	0.98 (0.93–1.05)	0.608	35	1.07 (0.93–1.24)	0.345
Prostatitis	8	0.99 (0.89–1.11)	0.895	15	1.04 (0.94–1.15)	0.488
Cathepsin X						
BPH	12	1.00 (0.96–1.03)	0.773	35	0.97 (0.86–1.10)	0.641
Prostatitis	12	1.08 (1.00–1.16)	0.0477	15	0.92 (0.83–1.03)	0.158
Cathepsin V						
BPH	7	0.94 (0.87–1.02)	0.160	35	0.97 (0.86–1.09)	0.615
Prostatitis	7	0.92 (0.75–1.13)	0.449	15	0.89 (0.80–0.99)	0.0349
Cathepsin D						
BPH	7	1.01 (0.97–1.06)	0.574	27	0.96 (0.83–1.12)	0.635
Prostatitis	7	0.97 (0.87–1.07)	0.526	7	1.03 (0.83–1.28)	0.755

The red font represents statistically significant positive results. MR, Mendelian randomization; BPDs, benign prostatic diseases; BPH, benign prostatic hyperplasia.

**Table 3:** Sensitivity analyses of MR analyses with a positive outcome.

Exposure	Outcome	P-value from Cochran’s Q test (IVW)	P-value from Cochran’s Q test (MR egger)	P value of pleiotropy test	P value of MR-PRESSO
Cathepsin O	BPH	0.535	0.452	0.785	0.579
Cathepsin X	Prostatitis	0.456	0.626	0.124	0.431
Prostatitis	Cathepsin V	0.290	0.238	0.707	0.335

In reverse MR, we found an association between genetically predicted prostatitis and cathepsin V levels by utilizing 15 prostatitis-related SNPs (**Supplementary Table 5**). Using IVW methods (**Supplementary Figure 5**), there is a decrease in the expression of cathepsin V in prostatitis cases (OR=0.89, 95% CI=0.80–0.99, p-value=0.0349). Similar to the analysis mentioned earlier, no signs of pleiotropy and heterogeneity were detected in the findings (P-value > 0.05) (**Table 3**; **Supplementary Table 6**; **Supplementary Figure 6**).

## Discussion

This study used MR to examine the reciprocal causal connections between cathepsins and BPDs. The results indicate that cathepsin O protected BPH, cathepsin X posed a risk for prostatitis, and prostatitis had a negative impact on cathepsin V. The findings provide valuable insights into the role of cathepsins in developing BPDs, potentially influencing the development of diagnostic and therapeutic strategies for cathepsins in patients with BPDs.

Previous research has indicated an association between cathepsins and PCa, which shows the possibility of clinical application value. Incorporating 474 males who had a prostate-specific antigen (PSA) concentration ranging from 2.0 to 10 ng/mL, a negative digital rectal examination, and an enlarged prostate (volume of at least 35 mL), a retrospective study revealed that combining thrombospondin 1, cathepsin D with %fPSA in a model could enhance the diagnosis of PCa and potentially decrease the need for unnecessary prostate biopsies. This model demonstrated improved specificity for PCa compared to using %fPSA alone (27). In PCa patients, a different protein expression test showed elevated levels of cathepsin S secreted by macrophages, indicating the progression of PCa. Another research by Jennica found that cathepsin H could regulate the migration of PCa cells (PC3) (28). Moreover, cathepsin E amplifies the effectiveness of doxorubicin against PCa cells in humans, which exhibit resistance to apoptosis induced by TRAIL. The up-regulation of cathepsin B promotes the invasion of PCa by producing sphingosine 1-phosphate by acid ceramidase (29). PCa is linked to the heightened presence of cathepsin B in plasma membrane/endosomal fractions, whereas BPH or normal prostate are not (30). Despite several studies suggesting potential applications of cathepsins in PCa disease, more studies need to explore cathepsins for BPDs. Importantly, this study is the initial MR to explore the correlation between cathepsins and BPDs, which warrants additional experimental and clinical investigation for validation.

There are multiple advantages to the current research. Initially, it was the primary study to investigate the impact of cathepsins on BPDs by utilizing extensive GWAS data from Finngen Biobank and the IEU OpenGWAS. Furthermore, since the chosen IVs were situated on a distinct chromosome, it is plausible that any potential gene-gene interaction would have minimal impact on the projected outcome. Furthermore, we employed various reliable techniques to acquire the MR effects, including MR-PRESSO and the Steiger test. Additionally, we assessed the existence of horizontal pleiotropy. By employing the two-step MR analysis, we successfully pinpointed the association between cathepsins and BPDs.

There are certain constraints in the current investigation. Initially, while three cathepsins were found to be associated with BPDs in our study, the effect was marginal and, probably, of limited clinical relevance. At present, there is a paucity of studies investigating the mechanism between cathepsins and BPDs. Previous studies have demonstrated that cathepsins are involved in all aspects of cellular activity and are strongly linked to the development of various diseases. Further research may yet reveal cathepsins to be a new diagnostic and therapeutic target for BPDs. Nevertheless, this hypothesis requires further experimental verification. Furthermore, the GWAS data may give rise to potential nonlinear relationships or stratification effects. Additionally, the findings might have restricted applicability to individuals of non-European origin because only participants with European heritage were included exclusively. This implies that the causal relationship postulated in this paper is applicable solely to European populations. The emergence of future GWAS data from other ethnic groups will be useful to further explore this causal inference. A comprehensive assessment of mixed populations will be more useful to explore the causal relationship between Cathepsins and BPDs at the genetic level. Lastly, given the complexity of genetic and environmental factors influencing BPDs and levels of cathepsins, we did not include possible confounding factors in our analysis. It is very difficult to identify confounding factors that affect both, and future studies should further identify confounding factors that can affect both to further improve the accuracy of results.

## Conclusion

To summarize, our findings indicate that cathepsin O played a beneficial role in BPH, cathepsin X posed a potential risk for prostatitis, and prostatitis had an adverse impact on the expression of cathepsin V. These cathepsins may become new targets for future BPDs diagnosis and treatment, but further validation is needed.

## Data availability statement

The original contributions presented in the study are included in the article/**Supplementary Material**. Further inquiries can be directed to the corresponding authors.

## Author contributions

HC: Writing – original draft, Supervision, Software, Formal analysis, Conceptualization. BL: Writing – review & editing, Software, Formal analysis, Data curation. KG: Writing – original draft, Software, Formal analysis, Data curation. HW: Writing – review & editing, Software, Formal analysis, Data curation. YW: Writing – review & editing, Software, Data curation. HZ: Writing – original draft, Software, Data curation. CS: Writing – original draft, Supervision. PW: Writing – review & editing, Supervision. HD: Writing – review & editing, Supervision. HZ: Writing – original draft, Investigation, Conceptualization. SW: Writing – review & editing, Investigation, Conceptualization.
