# Supplementary material for: Association between cathepsins and benign prostate diseases: a bidirectional two-sample Mendelian randomization study
Source: Front Endocrinol (Lausanne). 2024 Jun 5;15:1348310. doi: 10.3389/fendo.2024.1348310 (PMC11188316; doi:10.3389/fendo.2024.1348310)
Supplement: Supplementary file 1 [file DataSheet_1.docx]

**A list of Supporting Information**

**Supplementary figure 1**: MR results between cathepsin O and BPH.

**Supplementary figure 2**: (a) Leave-one-out between cathepsin O and BPH; (b) Funnel plot between cathepsin O and BPH

**Supplementary figure 3**: MR results between cathepsin X and prostatitis

**Supplementary figure 4**: (a) Leave-one-out between cathepsin X and prostatitis; (b) Funnel plot between cathepsin X and prostatitis

**Supplementary figure 5**: MR results between prostatitis and cathepsin V.

**Supplementary figure 6**: (a) Leave-one-out between prostatitis and cathepsin V; (b)Funnel plot between prostatitis and cathepsin V.


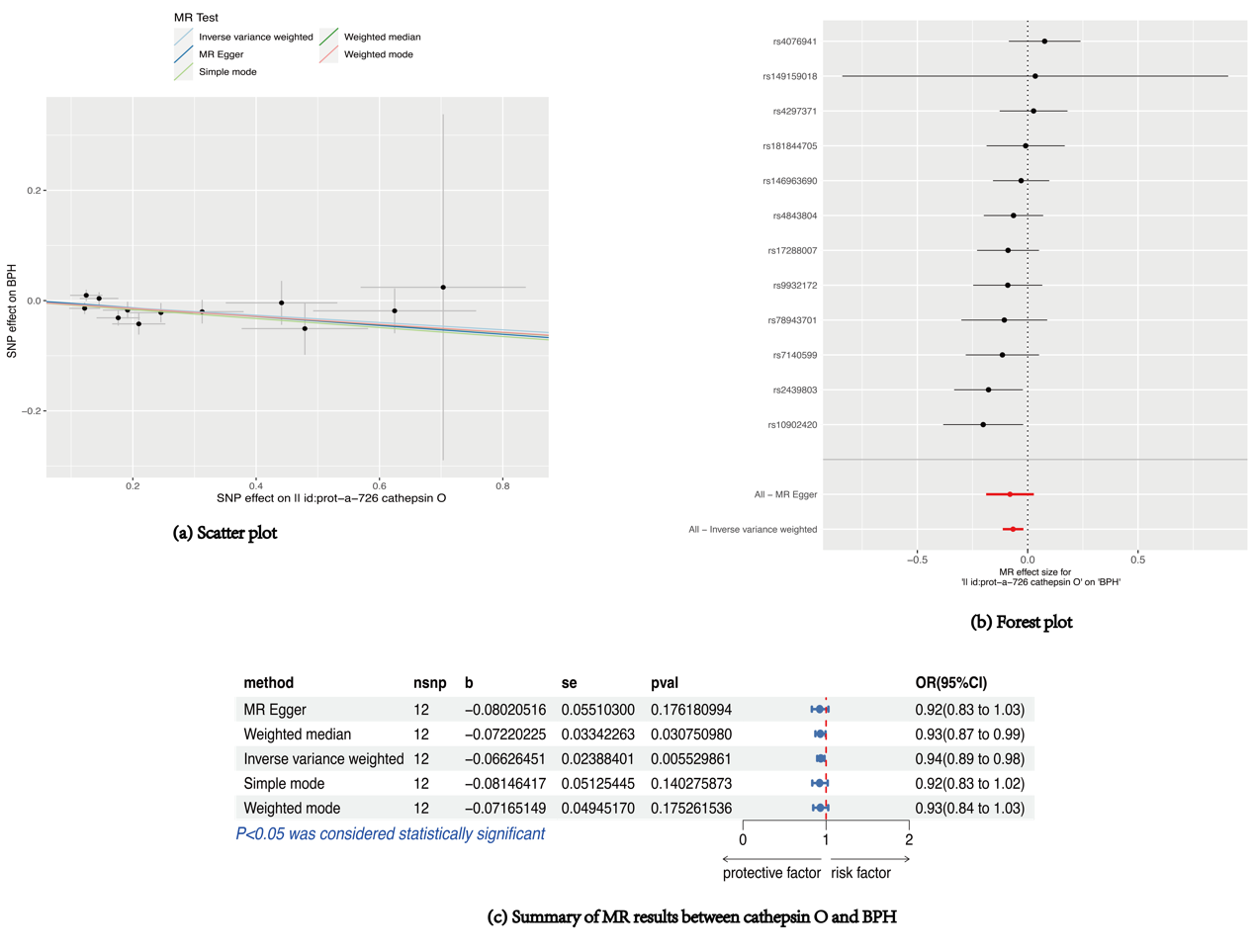


**Supplementary figure 1** **MR results between cathepsin O and BPH**. Abbreviations MR: Mendelian randomization; BPH: benign prostate hyperplasia.


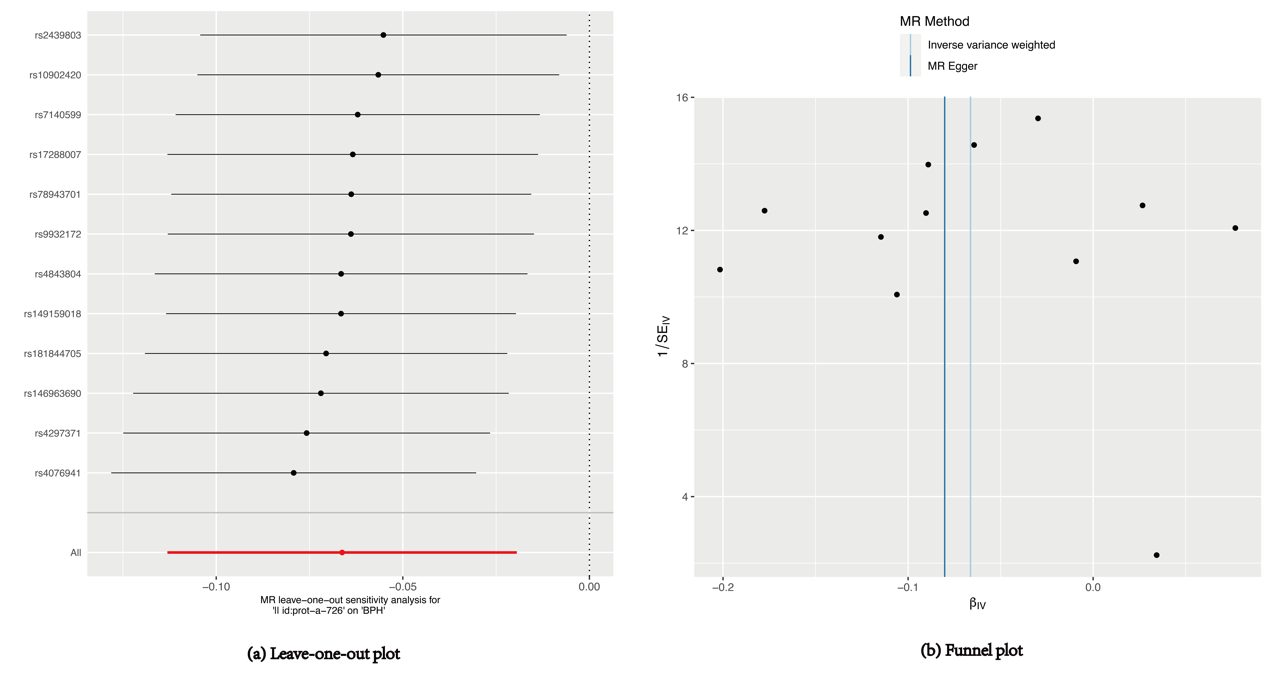


**Supplementary figure 2** (a) Leave-one-out between cathepsin O and BPH; (b) Funnel plot between cathepsin O and BPH. Abbreviation BPH: benign prostate hyperplasia


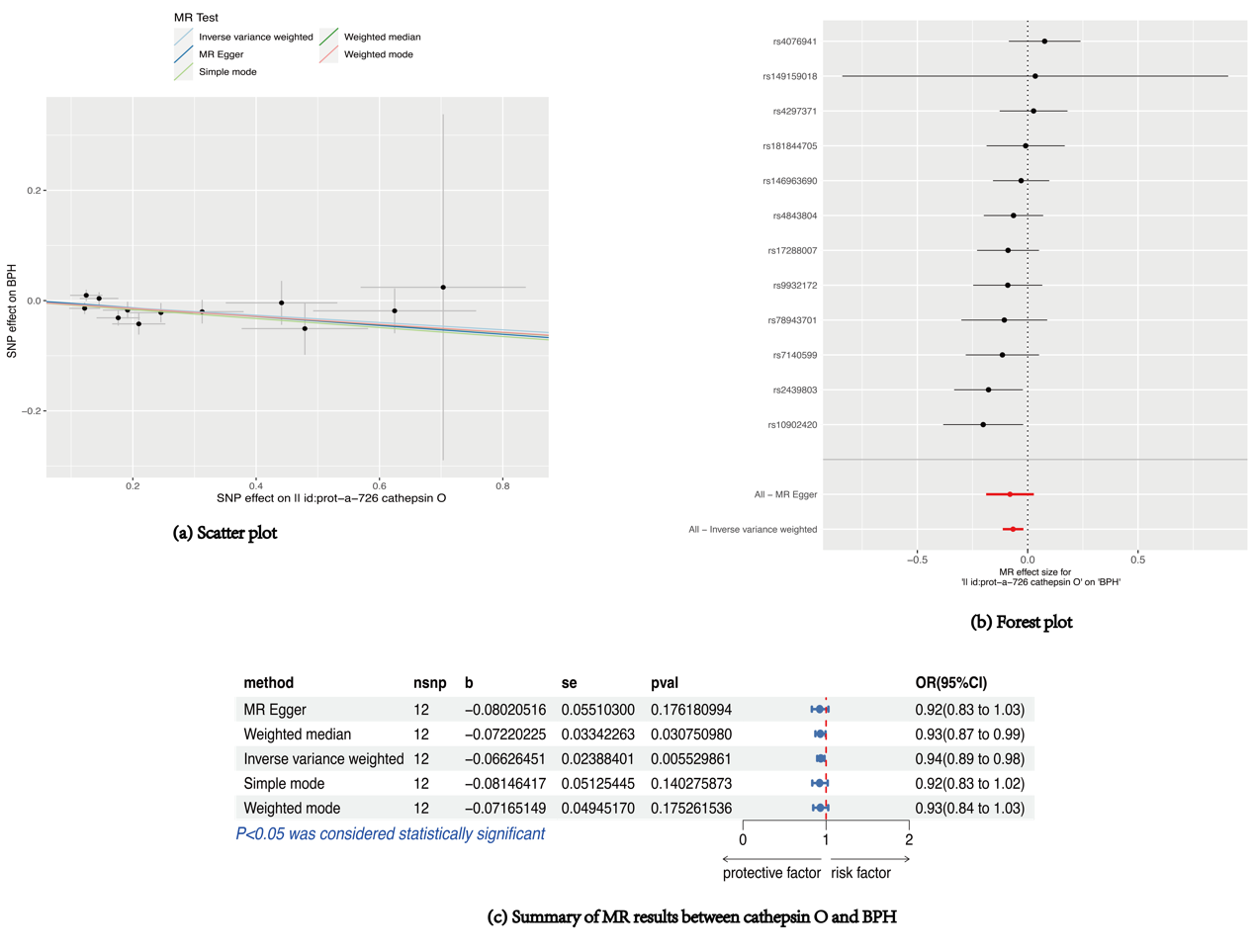


**Supplementary figure 3** **MR results between cathepsin X and prostatitis**. Abbreviations MR: Mendelian randomization.


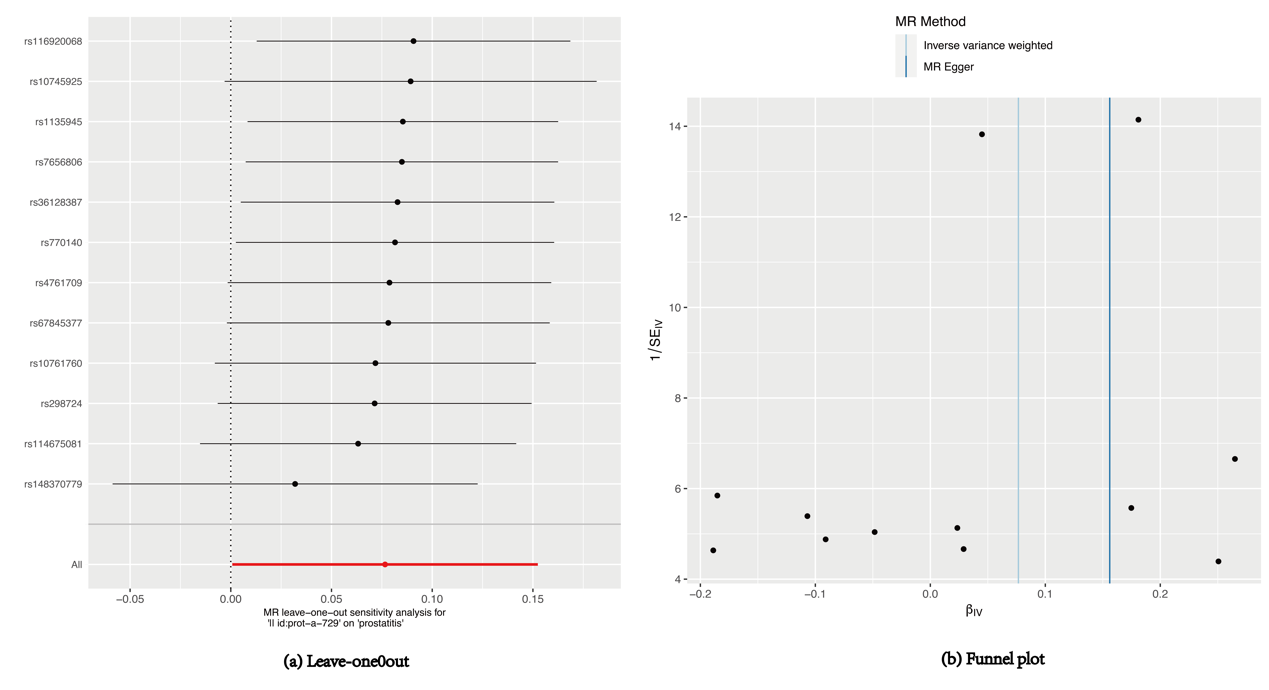


**Supplementary figure 4** (a) Leave-one-out between cathepsin X and prostatitis; (b) Funnel plot between cathepsin X and prostatitis


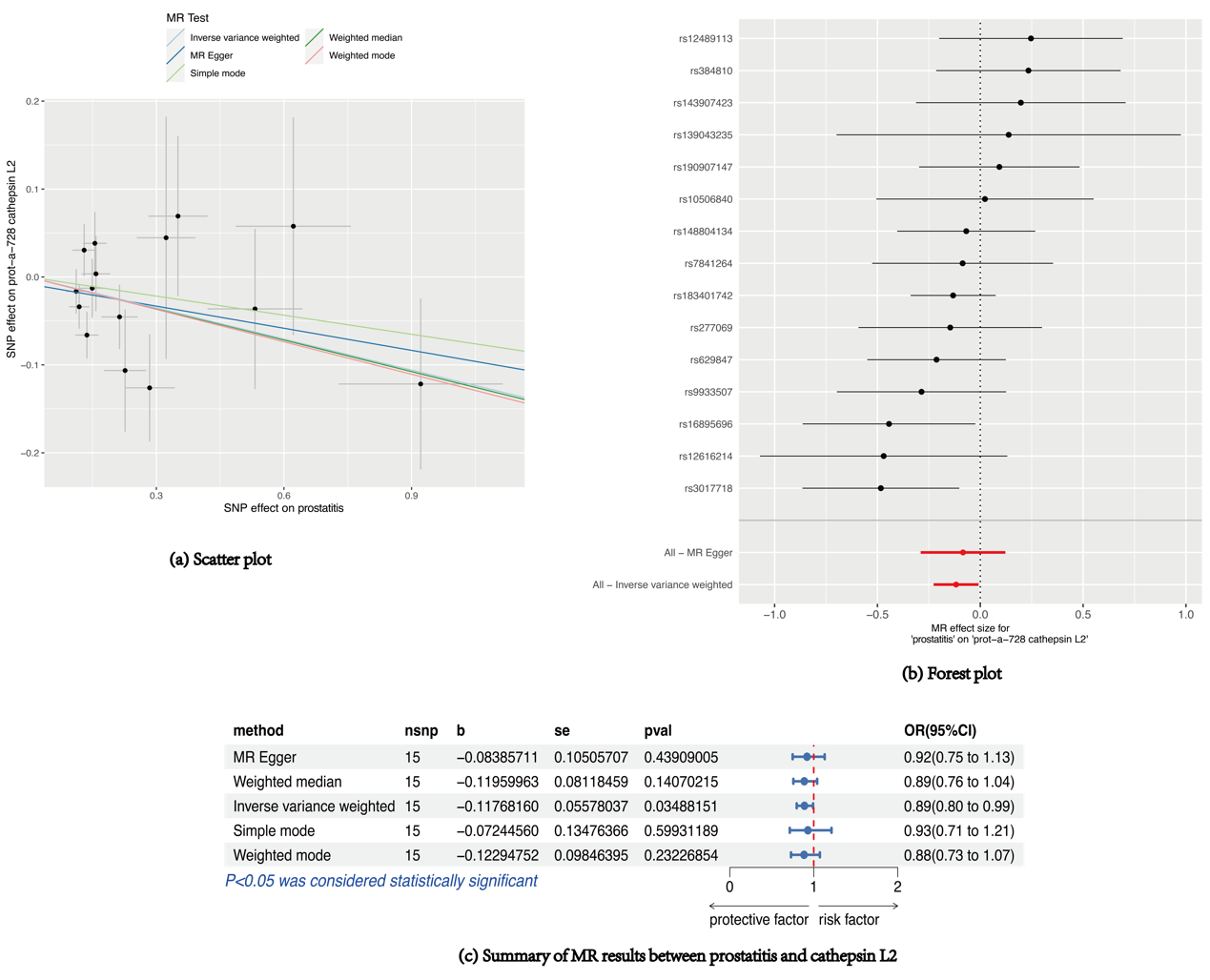


**Figure 5** **MR results between prostatitis and cathepsin V**. Abbreviations MR: Mendelian randomization.


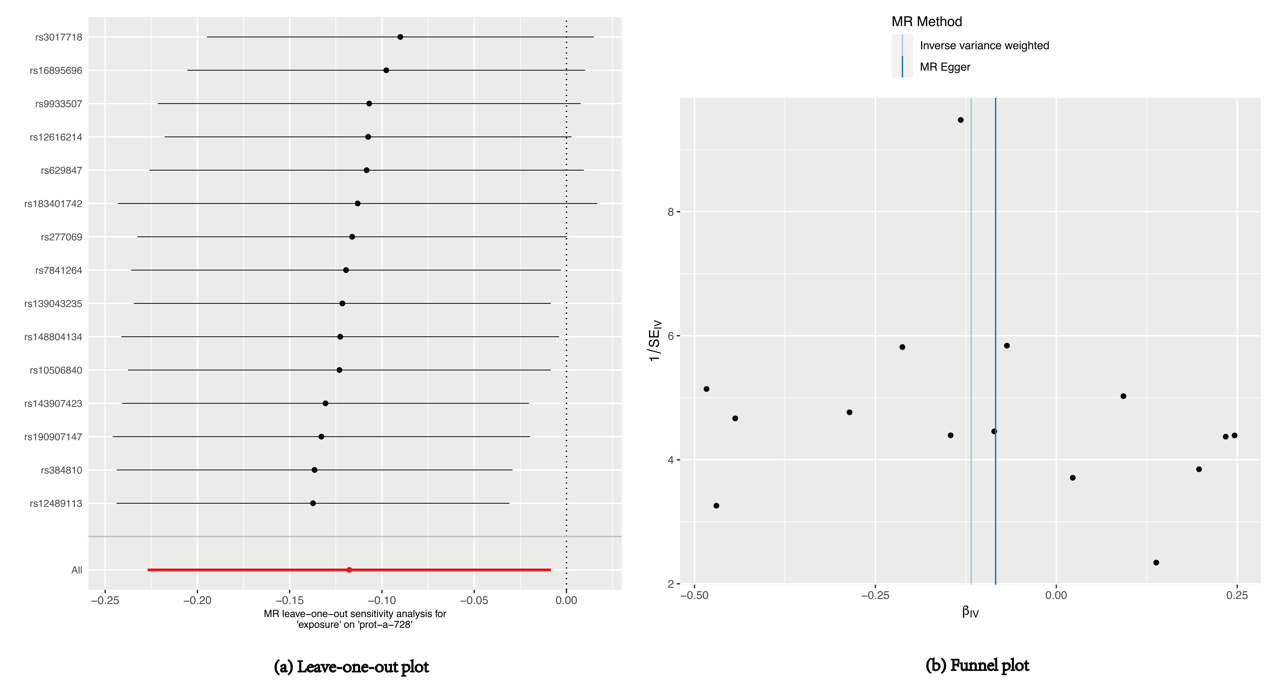


**Supplementary figure 6** (a) Leave-one-out between prostatitis and cathepsin V; (b)Funnel plot between prostatitis and cathepsin V.
